# Capturing the Experiences and Challenges of Emerging Adults in College During the COVID-19 Pandemic

**DOI:** 10.7759/cureus.17605

**Published:** 2021-08-31

**Authors:** Emily Hotez, Candace M Gragnani, Priyanka Fernandes, Kashia A Rosenau, Apsara Chopra, Ada Chung, Julie Grassian, Sydney Huynh, Tayloneei Jackson, Kevin Jimenez, Eric Jue, Nancy Le, Jennifer Lenghong, Alejandrina Lopez, Lizzet Lopez, Pearl Omo-Sowho, Kennedy Pennington, Richard Tirado, Alice Kuo

**Affiliations:** 1 Department of Internal Medicine, David Geffen School of Medicine, University of California, Los Angeles, USA; 2 Department of Preventive Medicine, David Geffen School of Medicine, University of California, Los Angeles, USA; 3 Department of Internal Medicine and Pediatrics, David Geffen School of Medicine, University of California, Los Angeles, USA; 4 Department of Medicine-Pediatrics, David Geffen School of Medicine, University of California, Los Angeles, USA

**Keywords:** covid-19, med-peds, emerging adulthood, health, college

## Abstract

Emerging adulthood (ages 18-30 years) coincides with “aging out” of pediatric care. As a result, combined internal medicine and pediatrics (Med-Peds) providers are tasked with promoting the health and well-being of this population during and post-coronavirus disease 2019 (COVID-19). In order to inform the response efforts, we aimed to capture emerging adults’ COVID-19 experiences and challenges during a two-week period of the pandemic in June 2020. We administered items from the California Health Interview Survey and an open-ended qualitative item via Qualtrics to 242 diverse emerging adults enrolled in a large US public university (mean age = 20.10, SD = 1.26). More than 90% of all students reported that they or their families or close friends experienced difficulties coping with the stressors and challenges presented by COVID-19. Almost half experienced financial difficulties and more than three-fourths experienced household disruptions during the pandemic. Qualitative findings underscored that these challenges were compounded by mental health and broader social issues. Recommendations for Med-Peds providers are provided to promote emerging adulthood health during and post-pandemic.

## Introduction

Emerging adulthood (ages 18-30 years) is a distinct developmental period characterized by heightened instability and susceptibility to a range of challenges. Given that this time coincides with the “aging out” of pediatric care, combined internal medicine and pediatrics (Med-Peds) providers are required to be attuned to these complex challenges in order to be responsive to their patients’ developmental needs and circumstances. During the coronavirus disease 2019 (COVID-19) pandemic, this task was particularly difficult due to the detrimental effects of the pandemic on emerging adults. For example, emerging adults were significantly more likely to contract the COVID-19 virus than was initially anticipated [1], with weekly COVID-19 cases among persons aged 18-22 years increased 55% nationally from August to September 2020 [2]. Further, emerging adults often delayed or avoided accessing routine and emergency healthcare services [3] and struggled with adhering to preventative health guidelines [4-6]. As a result, now more than ever, emerging adults are an at-risk population.

To address the needs of this population, Med-Peds providers must consider the key transitions that characterize emerging adulthood as a developmental period. Transitions in emerging adulthood pertain to health care, finances, living situations, and gaining independence. These transitions are often accompanied by substantial conflict, for example, although emerging adults are increasingly expected to autonomously manage their finances, they are often ill-equipped to succeed in these capacities given inadequate socialization, education, and skill-building prior to emerging adulthood [7]. These transitions are impeded by co-occurring developmental challenges in emerging adulthood, including increased vulnerability to mental health issues. For example, emerging adults have increased rates of depression, anxiety, non-suicidal self-injury, suicidal ideation, and suicide attempts [8]. Of particular concern is that many who require mental health services, supports, or treatments do not receive them, which further worsens mental health [9,10]. Racial and ethnic discrimination has been found to even further exacerbate mental health challenges in diverse emerging adults and has long been understood as detrimental to their sense of belonging, mental health, and educational success [11,12]. Importantly, more than 40% of emerging adults aged 18-24 years are currently pursuing a college education [13], which may further exacerbate these and other challenges. For example, college student debt has been found to have a deleterious effect on the subjective well-being of emerging adults [14]. Emerging adults often graduate with a number of complex health challenges, including struggles with increased substance use that often characterizes their college experiences [15].

Despite accumulating evidence for the need to support emerging adults, health-promoting interventions have, to date, remained insufficient for buffering against the detrimental effects of the pandemic [16]. Thus, it is critical to understand the impact of the COVID-19 pandemic on emerging adults in light of all of these factors. Research in this area will enhance Med-Peds providers’ capacities to translate findings into improvements in patient care.

The current study

This research presents findings from an online survey that captured the multi-faceted experiences of emerging adults in college in June 2020, a period that has been documented in the literature as a time following a month of high pandemic-related stress [17]. The current study aims to quantitatively and qualitatively describe the COVID-19-related, financial, environmental, and other-self-identified challenges faced by diverse emerging adults in college. Recommendations for Med-Ped providers are outlined.

## Materials and methods

This research involved the administration of an online Qualtrics survey at a large diverse public university over a two-week period in June 2020. Upon receiving a link to participate in the survey, participants completed two screening questions to ensure that they were at least 18 years of age and undergraduate students at the host institution. This research was co-developed with the Pathway for Students into Health Professions (PSHP) program, a program that promotes the development of a culturally diverse and representative healthcare workforce by recruiting and training students from underrepresented minority backgrounds interested in health professions. Researchers collaborated with the PSHP students (n = 14) to ensure that the research captured the most salient experiences, perspectives, and challenges of emerging adults.

Sample

Recruitment efforts targeted undergraduates attending a public university in Los Angeles, California with 31,382 undergraduate students enrolled as of 2018-2019 utilizing a multi-step collaborative convenience sampling method. The link to the survey was initially distributed via email to researcher- and PSHP-affiliated student networks. A snowball sampling strategy was adopted thereafter, such that the link could be distributed to as many students as possible. Although this method made it difficult to ascertain the response rate, it was particularly conducive to conducting research during the pandemic because it was convenient, free of cost, and yielded data quickly.

Demographic characteristics are presented in Table 1. The sample (n = 242) was representative of the host institution with respect to race (Asian/Native Hawaiian/Pacific Islander = 40.91%; White = 27.69%; two or more races = 8.68%; Native American/Alaska Native = 1.24%; Black/African American = 0.83%), ethnicity (Latinx = 14.05%), and non-US born (12.81%). On average, the sample was 20-years-old (mean [M] = 20.10, SD = 1.26) and predominantly female (68.60%). At the time of the survey, three-fourths reported living with their parents/guardians/families (73.55%).

**Table 1:** Demographic characteristics COVID-19, coronavirus disease 2019; ASD, autism spectrum disorder; ADHD, attention deficit hyperactivity disorder.

Demographic characteristics	Frequency (n = 242)	%
Race		
White	67	27.69%
Black/African American	2	0.83%
Asian/Native Hawaiian/Pacific Islander	99	40.91%
Native American/Alaska Native	3	1.24%
Two or more races	21	8.68%
No response	50	20.66%
Latinx	34	14.05%
Born outside of the US	31	12.81%
Female/trans woman	166	68.60%
Neurodivergence		
ASD	3	1.24%
ADHD	6	2.48%
Chronic illness	13	5.37%
Psychological disorder	39	16.12%
Learning disability	2	0.83%
Physical disability	3	1.24%
Other	3	1.24%
No response	20	8.26%
Parental income		
Less than $25,000	15	6.20%
$25,000-$74,999	28	11.57%
$75,000-$124,999	58	23.97%
$125,000-$249,999	54	22.31%
$250,000 or higher	29	11.98%
No response	58	23.97%
Living with parents pre-COVID-19	4	1.65%
Living with parents currently	178	73.55%
	Mean	SD
Age (years)	20.10	1.26

Measures

The survey development process first involved engaging PSHP students in research methods workshops and interactive opportunities to learn about principles of survey development. Following this, the first author led the PSHP students in collaborative virtual brainstorming meetings and internal pilot-testing. The final survey was based on informal consensus among the PSHP students regarding the most salient domains and items. The final survey assessed demographics and COVID-19-related, financial, and environmental challenges. We captured these challenges by utilizing items adapted from the adult version of the 2020 California Health Interview Survey (CHIS) [18] and an open-ended qualitative item. All multi-response quantitative items allowed participants to select all items that applied unless otherwise specified.

COVID-19-Related Challenges

These challenges were assessed by two sets of questions from the CHIS. The first utilized a five-point Likert scale (strongly disagree to strongly agree) for two items: “I have experienced difficulties trying to effectively cope with the numerous stressors/challenges presented by COVID” and “My family members and/or close friends have experienced difficulties trying to cope with the numerous stressors/challenges presented by COVID.” The second asked, “Have you experienced any of the following situations because of the COVID-19 outbreak?” Items were: “I've had to live with someone or care for someone diagnosed with COVID-19” and “I've continued to report to work because I was an essential worker.”

Financial and Environmental Challenges

Financial challenges assessed were food insecurity and general financial challenges experienced during the pandemic, utilizing items from the CHIS. Food insecurity items included two yes/no items: “After the onset of the pandemic, the food I bought didn't last and I didn't have money to get more” and “After the onset of the pandemic, I worried about whether my food would run out before I received money to buy more.” Other financial challenges were assessed through the question, “Have you experienced any of the following situations because of the COVID-19 outbreak?” and the options were: “I've lost my regular job,” “I've had a reduction in hours or a reduction in income,” “I've had an increase in childcare expenses,” “I've had financial difficulties with paying rent or mortgage,” “I've had financial difficulties with basic necessities, such as paying bills, affording groceries, etc.,” and “I've become uninsured because I cannot afford health insurance.” Environmental challenges were assessed through two questions, also from the CHIS. The first question was, “During the stay-at-home orders connected to the COVID-19 outbreak, was there an increase in your household of any of the following?” Items included: “Interpersonal conflict with family members or loved ones,” “Snapping at or yelling at family members or loved ones,” “Physical punishment of family members or loved ones,” and “Intrusive or interrupting noise.” The second question requested information about participants’ broader environments by asking whether they had been treated unfairly because of their race/ethnicity during the pandemic (yes/no).

Open-Ended Responses

Participants were given the option to respond to the question, “Please list any other challenges that you are experiencing right now due to COVID that have not been listed.” In order to ensure that the qualitative coding scheme for this question reflected the perspectives and priorities of emerging adults, the coding process was iterative, participatory, and collaborative with the PSHP students.

The first author developed a preliminary coding scheme based on an initial scan of recurring patterns and themes within the responses. At the same time, the first author led two qualitative coding workshops with the PSHP students focused on general principles and practical applications of qualitative analysis. Subsequently, students worked in pairs to critique, revise, pilot-test, and refine the preliminary coding scheme over a series of virtual meetings. It was collectively decided to align the final coding scheme with the organization of the quantitative items (i.e., COVID-19-specific, financial, environmental, and other challenges [which focused on mental health and social issue challenges]) to maximize the potential to integrate the findings. Within each category, students assigned codes if a recurring theme emerged three or more times within the data. Four PSHP students worked in two groups of two coders to code all of the quotations. One group focused on the codes pertaining to COVID-19-specific, financial, and other challenges; the other focused on codes pertaining to environmental challenges. Percent agreement for each code - determined by dividing the number of instances of agreement over the total number of opportunities for agreement - ranged from 92.22% to 98.70%.

## Results

All challenges and experiences are reported in Table 2. With respect to COVID-19-related challenges, more than 90% of all participants reported that they - or their families or close friends - experienced difficulties coping with the stressors and challenges presented by COVID-19. Few were continuing to report to work as an essential worker (3.72%) or living with or caring for someone diagnosed with COVID-19 (2.07%).

**Table 2:** Challenges and experiences during the pandemic among emerging adults in college (CHIS) COVID-19, coronavirus disease 2019; CHIS, California Health Interview Survey.

Challenges and experiences	Frequency (n = 242)	%
COVID-19-specific		
Experienced difficulties coping with stressors from COVID-19	225	92.98%
Family/close friends experienced difficulties coping with stressors from COVID-19	228	94.21%
Continued to report to work as an essential worker	9	3.72%
Live with or care for someone diagnosed with COVID-19	5	2.07%
Financial		
Worry about food running out	35	14.46%
Food did not last and not enough money to get more	16	6.61%
Experienced any financial difficulty below during the pandemic	107	44.21%
Reduction in hours or income	50	20.66%
Difficulties with basic necessities	35	14.46%
Job loss	34	14.05%
Difficulties with paying rent or mortgage	31	12.81%
Become uninsured/cannot afford health insurance	3	1.24%
Increase in childcare expenses	2	0.83%
Environmental		
Unfair treatment due to race/ethnicity	43	17.77%
Experienced any disruption below during the pandemic	190	78.51%
Interpersonal conflict with family members or loved ones	130	53.72%
Snapping at or yelling at family members or loved ones	143	59.09%
Physical punishment of family members or loved ones	13	5.37%
Intrusive or interrupting noise	149	61.57%

As to financial challenges, 14.46% reported that after the onset of the pandemic, they worried about whether their food would run out before they received money to buy more. Of the respondents, 6.61% reported that after the onset of the pandemic, the food they bought did not last and they did not have money to get more and 44.21% of the sample reported experiencing at least one of the financial difficulties assessed. The most common was a reduction in hours or income (20.66%), followed by difficulties with basic necessities, such as paying bills and affording groceries (14.46%), the loss of a regular job (14.05%), and difficulties paying rent or mortgage (12.81%). As to environmental challenges and experiences, 78.51% experienced an increase in at least one household disruption, including intrusive or interrupting noise (61.57%), interpersonal conflict (53.72%) or snapping or yelling at (59.09%), and physical punishment of family members or loved ones (5.37%). A total of 17.77% of students reported experiencing unfair treatment due to their race/ethnicity.

Qualitative themes and example quotations from the 96 respondents to the open-ended question are provided in Table 3. The most frequent COVID-19-related theme was concern about contracting COVID-19 (self and/or family; 14.58%), e.g., “I have an immunocompromised family member whose health is always a significant concern…” As to financial challenges, 13.54% reported finance, employment, and housing issues during the pandemic. These included financial challenges in the home, e.g., “recent unemployment of family members,” and broader trends in the US, e.g., “women are disproportionately losing jobs.” With respect to environmental challenges, several participants reported unhealthy, hostile, or counterproductive living situations (11.46%). They reported difficulties “trying to manage life from home,” and lack of study space in their homes. Additional themes emerged in the qualitative responses. These included general health concerns (22.92%, e.g., “I've been self-isolating because I'm paranoid I could have it and don't want my family to get sick in case I do…” They also included concerns about social interactions and relationships (15.63%), e.g., “touch starvation” and “loneliness.” Mental health challenges, in particular, emerged, including depression (10.42%) and difficulty getting help (4.17%), e.g., “my mental health is in shambles.” Social issue challenges included experiences with racism, ongoing protests, violence, and/or lack of safety (9.38%), including reports of gun violence. They also included frustration with news/media/politics (4.17%) and international student issues (2.08%).

**Table 3:** Qualitative challenges and experiences during the pandemic among emerging adults in college (n = 96) COVID-19, coronavirus disease 2019; PPE, personal protective equipment; EMT, emergency medical technician; BLM, Black Lives Matter.

Challenges and experiences	%	Example quotations
COVID-19-specific		
Concern/worry about contracting COVID-19 (self and/or family)	14.58%	A family member with who I am not living was tested for COVID-19. She stays in a hospital because she has a respiratory disease and is very at-risk. It was difficult to get information to her and help her from far away…this put massive stress on my family particularly my mother who is close to her.
		I have an immunocompromised family member whose health is always a significant concern…
Respondent or close family/friend is an essential worker	5.21%	Constantly worrying that one of my family members will accidentally contract it from working in the nursing facilities/hospitals. Worrying about their safety and insufficient supply of PPE.
		As an EMT working with COVID-19 patients, sometimes I’m worried about contracting COVID-19…
Financial		
Finance, employment, and housing challenges as the source of stress	13.54%	Added stress due to the recent unemployment of family members.
		Women are disproportionately losing their jobs compared to their male counterparts.
Environmental		
Living situations, including unhealthy/hostile living situations as the source of stress	11.46%	…trying to figure out how to manage life from home.
		I do not have access to designated study space in my home and can’t go out to the library etc. to get certain resources.
Other - mental health		
Health as a source of stress	22.92%	I’ve been having many arguments with family and friends over social distancing and being ridiculed for still adhering strictly to social distancing increased anxiety over COVID-19 due to a family member having serious surgery soon.
		I don't feel as productive as I normally would be at school. I've been self-isolating because I'm paranoid I could have it and don't want my family to get sick in case I do so I've lost 8 pounds because I don't eat as much.
Social interactions and relationships as sources of stress	15.63%	Touch starvation and social isolation are both challenges I am experiencing. I have not been able to see or hug my friends (or make any physical contact with them at all) in months and it is taking a toll on me. It feels like the only thing I have is my work for school and it is just a drag.
		Loneliness anxiety
Experiencing depression	10.42%	I’m depressed and can't find any motivation to do my school work.
		My mental health is in shambles and doing classwork seems like an impossible task.
Difficulty getting help	4.17%	The collective trauma of being removed from support systems at school such as friends, clubs/organizations, and therapy. Isolation has greatly increased my anxiety and depression and made it much more difficult to complete academic tasks.
		It’s been impossible for me to get access to mental healthcare. I suffered from untreated depression and anxiety prior to the pandemic. But now the pandemic has worsened my condition and it has been even harder for me to get into a therapy program. I tried telemedicine but it isn’t comprehensive enough…
Other - social Issue		
Experiences with racism, protests, violence, and/or lack of safety	9.38%	Various gunshots have risen in my area. The struggle for my parents to pay for financial needs has caused my alcoholic father to build an outrageous relationship with my mother and others around him. It is difficult to have a quiet space for testing. Many people in my area do not wear masks…
		Struggling with social distancing during BLM protests.
Frustration with news/media/politics	4.17%	I think the discrepancies between governments in different states/counties create a huge gap in freedoms and opportunities, which is problematic for an infinite number of reasons.
		COVID-19 coupled with the protests against racism that are shaking this country and bringing us to a monumental and historic moment that will be talked about in history in a good way or if things fail in a bad way.
International student issues	2.08%	I’m an international student in the US stuck. No flights are going home. I don’t know how long I’m going to be here for and my family is worried sick about my mental health, physical health, and safety. My lease ends June 30 so I have to move out but I don’t know where to go.
		Being an international student and sticking to deadlines/exams at US time.

## Discussion

Med-Peds providers require an understanding of the impact of the COVID-19 pandemic on emerging adults (ages 18-30 years). The current study presented findings from an assessment of diverse emerging adults attending a public university during a two-week period of the COVID-19 pandemic. This research captured a range of challenges and experiences utilizing both quantitative and qualitative data. The research methods were developed in collaboration with emerging adults from under-represented minority backgrounds. This research provided a multi-faceted understanding of their experiences and challenges during the pandemic, accompanied by corresponding recommendations for Med-Peds providers.

The first set of findings pertains to emerging adults’ experiences and challenges grappling with the pandemic itself. Indeed, almost the full sample experienced difficulties coping with the stressors and challenges presented by COVID-19. The qualitative data suggested that these difficulties are profound and pervasive, putting a “massive stress” on individuals and families, and leading to participants “constantly worrying” about the virus. While most reported concern with contracting COVID-19, they also reported a confluence of additional financial and environmental challenges. With respect to financial challenges, the proportion with food insecurity (6-14%) was on par with some reported national statistics [19]. Given the high proportion of Asian students in our sample, these findings should be interpreted in light of previous research that finds Asian groups to under-report financial insecurity on the CHIS [20]. Future research should make a concerted effort to better understand the financial challenges of Asian college students, particularly in light of the racism and discrimination Asian students may have experienced during the pandemic. Based on previous research, immigration status should also be factored into future research [21]. This line of inquiry is a critical area of future research given that food insecurity on the CHIS has been correlated with adverse health outcomes [22]. In addition, we found that more than 40% of respondents experienced any financial difficulties, and many respondents experienced stress and anxiety from finance, employment, or housing issues. Qualitative responses supported that students’ experiences of financial challenges were amplified by the pandemic, e.g., “Added stress due to recent unemployment of family members.” Future research should more directly investigate longitudinal financial trends among college students post-COVID-19, particularly among subgroups.

With respect to these environmental challenges, more than three-fourths experienced some type of household disruption, including intrusive or interruptive noise and interpersonal conflict. This finding is critical in light of research that finds household chaos to directly affect mental and physical health [23]. Indeed, there is consistent evidence for associations between household chaos and a number of adverse outcomes [24]. Household chaos often stems from financial instability, underscoring the inter-relatedness of all of the domains explored as part of this research [25]. Future research on emerging adults should explore the potential long-term consequences of household disruptions and chaos on health during the quarantine and stay-at-home orders.

Compounding these challenges, participants noted a number of additional stressors. For example, participants - comprised substantially of Asian and Latinx emerging adults - reported grappling with the recent rise in stigma, discrimination, and health disparities for racial and ethnic minorities. This was reflected in the quantitative data as well as in qualitative responses about racism, protests, and frustration with politics. Indeed, this comports with the social and political unrest that accompanied much of the pandemic. The qualitative data also underscored that the sample experienced marked challenges related to mental health. They reported depression, stress, and anxiety (e.g., “my mental health is in shambles…”). They described challenges with receiving help or support for their mental health challenges and pronounced feelings of isolation. Importantly, these two challenges are linked. Given that minority emerging adults have been found to be less likely to have formal mental health diagnoses - and are more likely to experience mental health challenges as a result of discrimination and stigma [26] - they often have significant untreated psychiatric problems [27]. As a result, emerging adults from minority backgrounds are at heightened risk.

Taken together, we propose the following recommendations for Med-Peds providers. These recommendations should be integrated with existing recommendations for working with emerging adult populations, including prioritizing building trust with patients, supporting patients in managing appointments, and ensuring that clinicians have the resources to effectively serve this population [28].

First and foremost, there is a resounding need to promote access to and uptake of mental health care. Almost all emerging adults in our study struggled with coping with the COVID-19 pandemic. A substantial proportion experienced financial instability, household disruptions, social unrest, and mental health challenges. All of these factors directly impact emerging adult health. As a result, Med-Peds providers should, to the fullest extent possible, facilitate patient access to mental healthcare services. These services should be flexibly offered, both remotely and in person, and accessible and available to low-income and marginalized groups. There should be a concerted effort to ensure that patients are aware of these supports. In practice, this means that providers should ensure that emerging adult patients receive timely mental health referrals and are readily able to act upon these referrals. This may entail provider collaborations and partnerships with social workers, hotlines, or volunteer organizations. It may also involve efforts on the part of Med-Peds office staff to facilitate appointment scheduling and follow-through by utilizing methods that are salient to emerging adults (e.g., text messaging). Efforts should be further tailored to specific sub-groups (e.g., college students and/or minority or historically marginalized groups) to maximize their efficacy for those with distinct needs.

The second recommendation goes hand-in-hand with the first: Med-Peds providers should promote multi-dimensional mental and physical health and well-being in healthcare interactions. In clinical settings, this may entail speaking about mental health as a critical component not separate from physical health. Assessments should include indicators of each. For example, providers may increase their utilization of standardized screeners for depression (e.g., the Patient Health Questionnaire-9 [PHQ9]). Providers should specifically ask about salient developmental challenges for emerging adults, including the presence of financial challenges, household disruptions, and other environmental stressors. Particular attention should be paid to historically marginalized groups, including racial and ethnic minorities. This may involve administering social determinants of health screener at all annual exams or chronic disease follow-up appointments to facilitate connections to community-based resources.

Finally, a significant strength of our research was in the collaboration with emerging adults themselves. Moving forward, physician training materials and patient resources should be developed, implemented, and evaluated utilizing a similar approach to ensure that they are effective and relevant for the emerging adult population. In practice, this entails forging partnerships with researchers who can lead quality improvement efforts that position emerging adults as collaborators in the process. These collaborations may result in the development of novel physician education and training resources that would facilitate their capacity to support this population. These resources could take the form of Continuing Medical Education (CME) courses and/or medical school or resident curricula. These collaborations may also result in the development of new practices, policies, and/or resources that would serve the emerging adult population.

The current study is not without its limitations. This research relied on data from a single point in time from a relatively small sample of emerging adults in college. Future research should employ a longitudinal design with larger samples to capture change over time and generate more conclusive findings. Additionally, although the qualitative data in our study provided important insights into the challenges and experiences among emerging adults in college, future research should utilize mixed methodologies (e.g., survey and interview data) to obtain more detailed qualitative data. Finally, the host institution has a particularly small Black/African American student population. It will be particularly important to capture Black/African American students’ experiences during and post-COVID-19 in future research.

## Conclusions

Emerging adults have experienced COVID-19-related, financial, environmental, mental health, and other challenges during the pandemic. These findings facilitate a comprehensive understanding of emerging adults’ complex experiences and challenges during the COVID-19 pandemic. This research supports Med-Peds providers’ efforts to promote holistic well-being for emerging adults during and post-pandemic.
